# Targeting Oxidative Stress Involved in Endometriosis and Its Pain

**DOI:** 10.3390/biom12081055

**Published:** 2022-07-29

**Authors:** Lauren Clower, Taylor Fleshman, Werner J. Geldenhuys, Nalini Santanam

**Affiliations:** 1 Department of Biomedical Sciences, Joan C Edwards School of Medicine, Marshall University, Huntington, WV 25755, USA; clower4@marshall.edu (L.C.); burke74@marshall.edu (T.F.); 2 Department of Pharmaceutical Sciences, School of Pharmacy, West Virginia University, Morgantown, WV 26506, USA; werner.geldenhuys@hsc.wvu.edu; 3 Department of Neuroscience, School of Medicine, West Virginia University, Morgantown, WV 26506, USA

**Keywords:** antioxidant, inflammation, ferroptosis, pain

## Abstract

Endometriosis is a common gynecological disorder seen in women and is characterized by chronic pelvic pain and infertility. This disorder is becoming more prevalent with increased morbidity. The etiology of endometriosis remains to be fully elucidated, which will lead to improved therapeutic options. In this review, we will evaluate the biochemical mechanisms leading to oxidative stress and their implication in the pathophysiology of endometriosis, as well as potential treatments that target these processes. A comprehensive exploration of previous research revealed that endometriosis is associated with elevated reactive oxygen species and oxidation products, decreased antioxidants and detoxification enzymes, and dysregulated iron metabolism. High levels of oxidative stress contributed to inflammation, extracellular matrix degradation, angiogenesis, and cell proliferation, which may explain its role in endometriosis. Endometriosis-associated pain was attributed to neurogenic inflammation and a feed-forward mechanism involving macrophages, pro-inflammatory cytokines, and pain-inducing prostaglandins. N-acetylcysteine, curcumin, melatonin, and combined vitamin C and E supplementation displayed promising results for the treatment of endometriosis, but further research is needed for their use in this population.

## 1. Introduction

Endometriosis is an underdiagnosed cause of chronic pelvic pain and infertility in up to 10–15% of reproductive-aged women [1,2]. It is defined by ectopic growth of endometrial tissue, most commonly in the pelvic peritoneum, ovaries, and rectovaginal septum [3,4]. While it is a benign diagnosis, it is often difficult to treat with histopathological hallmarks characteristic of cancer. Endometriosis can drastically impact quality of life as it can present from menarche to menopause. Risk factors include early menarche, late menopause, and nulliparity, all of which increase the number of lifetime ovulatory cycles [5]. Common clinical symptoms include irregular uterine bleeding, painful intercourse (dyspareunia), and painful periods (dysmenorrhea) [6,7]. As there is no cure for endometriosis and the pathophysiology remains unclear, common treatment options target relieving symptoms but do not address the underlying mechanisms of the disease [8]. A reduction in estrogen production and ovulation suppression are often targeted by treatments to alleviate pain and slow disease progression, yet surgery is often utilized for refractory cases or infertility [9,10].

Current theories for the origin of endometriosis include coelomic metaplasia, Müllerianosis, extrauterine stem cell differentiation, benign lymphatic or hematogenous metastasis, bacterial contamination, and the most plausible theory, Sampson’s retrograde menstruation [10,11,12]. Current investigations agree that genetic predisposition, inflammation, and estrogen dependence are all hallmarks of the disease [10,11]. Endometriosis is primarily associated with chronic pelvic pain derived from macrophage and mast cell activation which contributes to a vicious cycle of persistent inflammation, oxidative stress, and pain [13]. Oxidative stress is a potential component of the pathophysiology underlying endometriosis and is characterized by an imbalance between reactive oxygen species (ROS) and antioxidants which results in an abundance of ROS and a deficiency of antioxidant mechanisms [13,14]. Oxidative stress also plays a role in endometriotic pain, yet current knowledge regarding its etiology is limited [1]. This prohibits the design or conception of efficacious treatment options for endometriosis or its associated pain in these patients. The goal of this review is to elucidate (i) the role of oxidative stress in endometriosis, (ii) the mechanisms underlying endometriotic pain, and (iii) novel treatment options for endometriosis-associated oxidative stress.

## 2. Endometriosis and Oxidative Stress

### 2.1. General Oxidative Stress

Reactive Oxygen Species (ROS) include both free radicals and non-free-radical oxygen intermediates which are generated endogenously. Free radicals, such as singlet oxygen (^1^O_2_), hydrogen peroxide (H_2_O_2_), superoxide (O_2_^•−^), hydroxyl radicals (OH^•^), and nitric oxide (^•^NO), are highly reactive due to the presence of unpaired electrons [15]. These molecules can directly interact with and change the composition of lipids, proteins, and nucleic acids which results in unstable lipid membranes, misfolded proteins, and DNA breakage [16,17]. As free radicals are produced during normal physiological processes, antioxidant defense mechanisms must be in place to prevent the accumulation of these reactive species. Endogenous antioxidant enzymes include superoxide dismutase (SOD), catalase, and glutathione peroxidase (GPx) [15,16,18]. SOD converts superoxide anions to the less reactive hydrogen peroxide, which is then converted to water by catalase or GPx. Other defenses of free radical-mediated damages include vitamins such as vitamins A, C, and E which stabilize free radicals by donating a hydrogen atom [16,18,19,20]. Oxidative stress is the imbalance between the levels of ROS and other oxidants and levels of antioxidant defense.

While excess ROS or oxidative stress are often considered for their harmful effects, they also have physiological purposes. They serve to amplify the body’s defense mechanisms during intense exercise or ischemia, help prepare the birth canal for delivery, and participate in signaling pathways [16,19,21,22,23]. Redox signaling also plays an integral role in cell migration, circadian rhythm, and stem cell proliferation [15,24]. Cells in the immune system utilize ROS to kill invading organisms such as the oxidative burst, which is used by phagocytes to degrade engulfed pathogens [25]. However, in addition to their ability to directly interact with biological molecules (lipids, proteins or nucleic acids), ROS damage also involves the alteration of signaling pathways, such as activation of the NF-κβ pathway, which in turn activates the gene expression of several pro-inflammatory cytokines [16,19,26]. NF-κβ is a transcription factor that becomes phosphorylated and then binds to DNA binding regions to induce the transcription of oncogenes [26,27]. This pathway can also function to induce genes such as NADPH oxidase NOX2 subunit gp91 phox which produces a subunit involved in the electron transport chain of oxidative phosphorylation [28,29]. This subunit is responsible for donating electrons from NADPH to molecular oxygen which generates superoxide anions (O_2_^•−^) and increases oxidative stress [28,29,30]. Oxidative stress is prominent in cancer, atherosclerosis, neurodegenerative disorders, and other inflammatory diseases [30,31,32,33,34,35,36,37].

To evaluate the role of oxidative stress in various diseases, it is necessary to measure markers of ROS. This can be accomplished by using general fluorescent probes such as dichlorodihydrofluorescein that detects all the ROS contained within a sample, or with probes that target a specific ROS using electron paramagnetic resonance spectroscopy [24]. Another option is to measure products of oxidation, including oxidized DNA, RNA, proteins, and lipids [24,38,39]. A very common method for detecting oxidative stress in biological samples is to measure thiobarbituric acid-reactive substances (TBARS) spectrophotometrically, which are representative of lipid peroxidation, or protein carbonyls, which are representative of protein oxidation [32,39].

### 2.2. Endometriosis and Oxidative Stress

The role of oxidative stress in the development and progression of endometriosis has been well-established by our studies [40,41,42,43,44,45,46,47,48] and other researchers who have confirmed the presence of elevated oxidative stress markers in comparison to control groups [49,50,51]. This relationship is particularly important as oxidative stress has been implicated in various disease processes and may be responsible for local tissue destruction in endometriosis [52]. Oxidative stress is characterized by the formation of ROS during endogenous oxygen metabolism. These ROS, namely superoxide and hydrogen peroxide (H_2_O_2_), are known to modulate cellular proliferation in endometriosis [15,16,53,54]. Cellular proliferation of various cell types such as endothelial, epithelial, or stromal cells is one of the customary hallmarks exhibited by endometriosis which promotes enhanced survival and implantation of these cells at ectopic sites [55,56,57].

Studies have examined the relationship between endometriosis and various markers of oxidative stress, including ROS, products of oxidation, detoxification enzymes, and antioxidants. While there seems to be an association between oxidative stress and endometriosis, studies have conflicting results regarding the presence of various oxidative stress markers. Levels of malondialdehyde (MDA), a TBARS, were significantly elevated in blood, serum, peritoneal fluid, and follicular fluid in endometriosis patients when compared to control groups [58,59,60]. Our studies found increased lysophophatidyl choline (a marker of lipid peroxidation), a chemotactic factor for macrophages [61] and autoantibodies to oxidative stress markers [41] in the peritoneal fluid of women with endometriosis. Another study found increased levels of serum iron, a producer of free radicals and oxidative stress [62], which was confirmed in a study that found elevated levels of ferritin, the storage form of iron, in endometriosis patients [63]. Recent studies have suggested that dysregulated iron homeostasis and excessive iron are implicated in endometriotic oxidative stress and may lead to peripheral ferroptosis [64,65]. Ferroptosis is discussed in detail later in this review.

Endometriosis patients have decreased serum thiol levels and decreased total antioxidant capacity, which increases their susceptibility to oxidative stress [54,60,66]. Santulli et al., found conflicting results of normal thiol levels, yet advanced oxidation products, nitrites, and nitrates were elevated in the peritoneal fluid that is indicative of increased oxidative stress [67]. Heat shock proteins such as HSP70 which have stress-inducible expression and function as protective agents against cellular oxidative stress and inflammation, are found at elevated levels at sites with lipid accumulation (oxidized low-density lipoprotein) and activated macrophages, such as during the development of atherosclerotic plaques [68]. HSP70b’, in particular, has been used as an indicator of oxidative stress in endometriosis at increased levels [69,70].

Several studies have proposed mechanisms as to how oxidative stress contributes to endometriosis by demonstrating the connection between oxidative stress, inflammation, and extracellular matrix (ECM) degradation. Elevated expression of various cytokines, growth factors, and matrix metalloproteinases (MMPs) are found in serum samples of women with endometriosis [71]. Similarly, Qiu et al., showed that high levels of IFN-γ and IL-10, precipitated by increased IL-2 and IL-27, promoted proliferation and invasion of endometrial stromal cells [72]. Cytokine IL-10, in particular, has an important role in the development of endometriosis as its expression results in the activation of MMPs, ECM remodeling, and angiogenesis [71]. Increased expression of IL-10 in serum and PF may be attributed to upregulated activation of the NF-κβ signaling pathway resulting from oxidative stress and iron overload in the peritoneal cavity (PC) [71,73]. The NF-κβ mediated transcriptionally activated oncogenes such as COX-2 repress apoptosis and promote cellular proliferation in endometriosis [27,74]. The net effect of inducing the NF-κβ pathway in endometriosis is increased expression of other inflammatory cytokines, inflammatory genes such as COX-2, and adhesion [27]. Downregulation of this transcription factor led to an improvement in endometriosis symptoms [75,76]. Oxidative stress also induces other pathways such as upregulation of glycodelin which increases VEGF expression and angiogenesis [44]. ROS-mediated activation of protein kinase ERK1/2 causes changes in cell proliferation and survival of endometrial cells, such as those seen in tumor cells [77]. Our studies have shown that oxidized lipoproteins that are abundant in the endometriotic PF can induce MCP-1 [78] and CSF-1 and its receptor c-fms in endometrial cells [79].

Researchers have demonstrated that disease severity in endometriosis often corresponds to the levels of oxidative stress markers. Advanced peritoneal oxidation products are indicative of disease severity [67]. Reduced activity of endogenous antioxidant enzymes such as SOD or GPx and increased accumulation of lipid peroxides may also correlate to more severe cases of endometriosis [59]. While HSP70b’ is a good measure of oxidative stress in endometriosis, it does not indicate disease severity [69]. Several studies have illustrated the etiological role for oxidative stress in endometriosis and its pain. The pharmaceutical agents and natural products shown to have beneficial effects in endometriosis also modulate oxidative stress pathways. Hence, assessing oxidative stress markers as a readout for the treatment efficacy of these agents might be useful.

### 2.3. Ferroptosis

**Figure 1:**
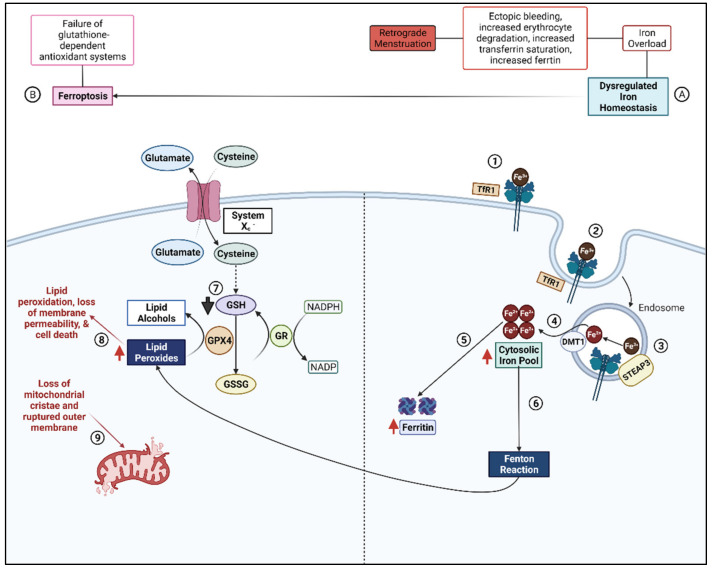
Dysregulated Iron Homeostasis Leads to Oxidative Stress and Ferroptosis in Endometriosis. (A) Retrograde menstruation, a proposed mechanism for disruption of iron homeostasis in endometriosis, induces ectopic bleeding in the peritoneal cavity (PC). This causes erythrocyte degradation which releases free iron. High levels of iron promote increased transferrin saturation. (1) Transferrin binds to iron and delivers it to tissues where it binds to the transferrin receptor (TfR1). (2) The TfR1-Fe3+ complex enters the cell in an (3) endosome where acidification enables the separation of ferric iron (Fe3+) from the receptor. Six transmembrane epithelial antigens of the prostate 3 (STEAP3) catalyzes the conversion of ferric iron to ferrous iron (Fe2+). (4) Ferrous iron leaves the endosome and accumulates in the cytosolic iron pool. (5) Increased levels of iron in the cytosolic pool leads to increased levels of the intracellular iron storage protein, ferritin. (6) A portion of excess iron will undergo the Fenton reaction to generate free radicals which can damage lipids, proteins, and DNA. (B) This schematic represents a simplified diagram of ferroptosis, an-iron dependent mechanism of cell death. Ferroptosis occurs when glutathione-dependent antioxidant systems fail due to the accumulation of excessive reactive oxygen species (ROS). (7) There is decreased availability of reduced glutathione (GSH) due to excessive intracellular ROS. GSH is required for the conversion of lipid peroxides to non-toxic lipid alcohols by the enzyme glutathione peroxidase (GPX4). (8) Lipid peroxides accumulate and reduce membrane permeability and (9) promote the loss of mitochondrial cristae which contributes to mitochondrial outer membrane rupture. The intracellular accumulation of lipid peroxides and decreased total antioxidant capacity (TAC) eventually leads to cell death. Adapted from “Role of Lipin-1 in Modified-LDL Induced Pro-inflammatory Response”, by Biorender.com (2022). Retrieved from https://app.biorender.com/biorender-templates (accessed on 18 July 2022).

**Figure 2:**
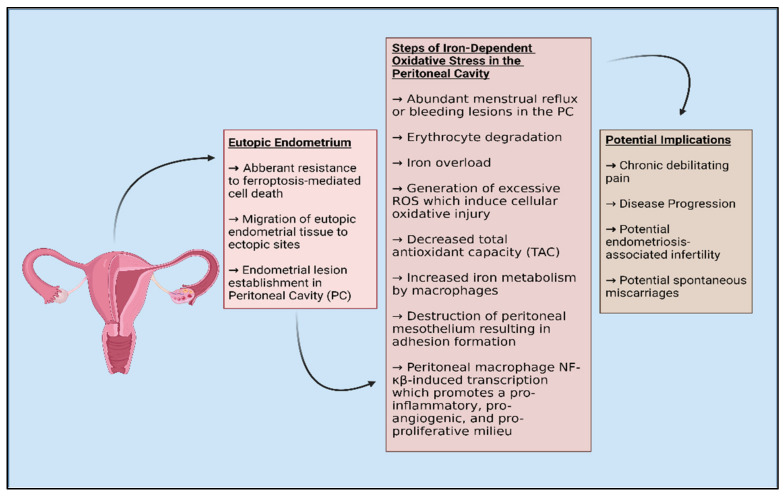
A simplified diagram of the etiology underlying iron-dependent oxidative stress characteristic of endometriosis.

Ferroptosis is a newly coined term for cell death, first introduced circa 2012 [80], and has recently been shown in endometriosis [81]. This new description of an ROS-mediated cell death mechanism has been investigated in several diseases, including cancers and neurodegenerative diseases (Figure 1). This molecular mechanism has gained traction in the past few years, with the possible design of ferroptosis inducers as novel anti-cancer agents, while ferroptosis inhibitors can act as novel neuroprotective agents. Cancer cells have adapted to survive with increased cellular ROS production during proliferation/hyperproliferation by upregulation of several antioxidant proteins [82]. With ferroptosis, the anti-oxidant mechanisms, specifically the glutathione-dependent systems, are inadequate to provide protection against the ROS, which leads to lipid peroxidation [80]. The lipid peroxidation in ferroptosis is due primarily to iron-mediated mechanisms, based on Fenton chemistry whereby iron catalyzes a free-radical reaction with polyunsaturated fatty acids (PUFA). Glutathione peroxidase 4 (GPX-4) is important for regulating the ROS and prevents the buildup of the lipid peroxides, by reducing hydrogen peroxide (H_2_O_2_). Glutathione (GSH) is necessary for the function of GPX-4. The synthesis of GSH is based on the availability of cysteine, which is transported by amino acid transporters, or cystine, the oxidized form of cysteine. Cystine is transported into cells via a x_c_^−^ cystine/glutamate antiporter in the GSH synthesis pathway. Inhibition of the x_c_^−^ cystine/glutamate antiporter leads to a decrease in the intracellular GSH levels. An example of the effects seen by inhibition of cystine uptake into cells is from the compound erastin [83]. Recently, the group of Li et al., showed that erastin is able to trigger ferroptosis in endometrial cells to a greater extent than the normal stromal cells as well as regression of endometriosis lesions in a mouse model [81]. This finding is further supported by a study which shows that ferroptosis resistance is important for endometriosis survival [64]. For eutopic endometriosis, ferroptosis favoring mitochondrial mechanisms is seen, while ectopic favors autolysosomal pathways [64] (Figure 2). Hence, ferroptosis is suggested to have a good biomarker/diagnostic value in endometriosis.

## 3. Endometriosis and Pain

**Figure 3:**
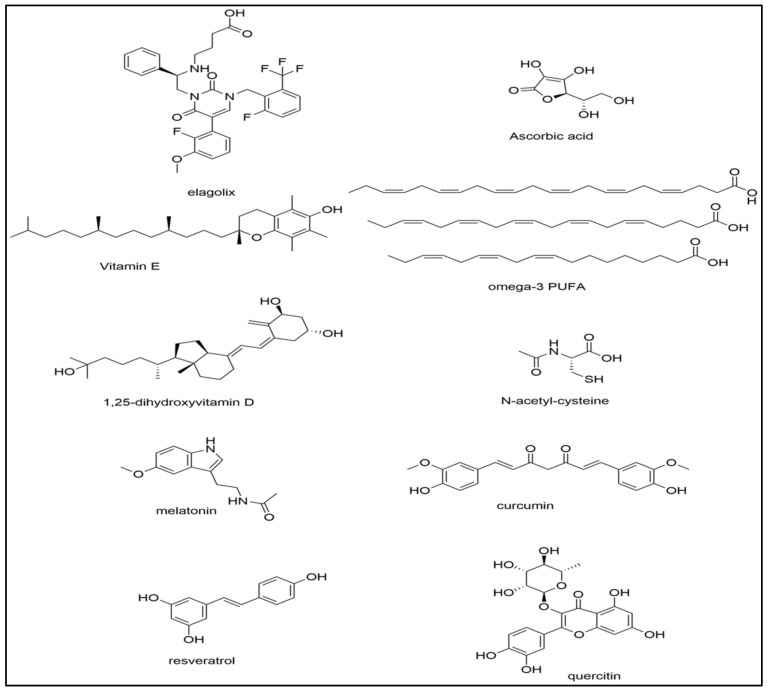
The chemical structures of several key compounds discussed in the review which have been used for the treatment of endometriosis.

Approximately 20–25% of women with endometriosis present clinically as asymptomatic [1]. For symptomatic patients, the most common symptom experienced is pelvic pain [84,85]. Other types of presenting pain include dysmenorrhea, dyspareunia, dyschezia, neuropathic pain, hyperalgesia, and allodynia [85,86]. Currently, knowledge is limited regarding the underlying mechanisms behind endometriotic pain, so effective treatment options are limited. The standard method to diagnose and excise ectopic pain-inducing endometrial lesions is surgical laparoscopy, which is an invasive procedure primarily used for refractory cases of pain and to improve fertility in women with endometriosis [87]. Unfortunately, this procedure must be utilized repetitively upon the recurrence of lesions and thus, is not the best treatment option. There are other less invasive treatment options available to alleviate endometriosis-associated pain such as oral contraceptives, gonadotropin-releasing hormone agonists (GnRH) (leuprolide), aromatase inhibitors, and analgesics such as non-steroidal anti-inflammatory drugs (NSAIDs) [86]. Elagolix, (Figure 3), is a small molecule compound that is a first-in-class GnRH receptor antagonist approved for the treatment of moderate or severe pain associated with endometriosis [88,89]. The discovery of elagolix as a non-peptide antagonist was first described with disclosure of the synthesis and biological evaluation of several uracil phenylethylamines which were substituted with butyric acid. Elagolix was found to bind to human GnRHR with a Ki of 0.9 nM, and inhibited GnRH stimulated inositol phosphate production with an IC_50_ of 1.5 nM and was found to be orally available [89].

Many of these treatments target estrogen dominance which is ordinarily associated with increased inflammation and chronic pelvic pain [90]. By reducing estrogen levels, these therapeutic options aim to relieve pelvic pain, yet pain-related symptoms often persist while taking these medications. Hence, there is an essential need to re-evaluate the mechanisms behind endometriosis-associated pain to develop more effective novel treatment options.

### 3.1. Mechanisms of Pain

It has been well-established that endometriosis-associated pain often results from aberrant inflammation and the dysregulation of inflammatory factors which is commonly seen in chronic autoimmune diseases such as irritable bowel disease (IBD) and rheumatoid arthritis (RA) [84]. Previous studies have suggested that macrophages, neurogenic inflammation, lipid peroxides, and pain-inducing prostaglandins play a vital role in the pathophysiology of endometriotic pain [46,91,92,93].

#### 3.1.1. Macrophages

Macrophages have a proposed role in the pathogenesis of endometriosis-associated chronic pain. During endometriosis, higher numbers of macrophages are recruited to the peritoneal cavity (PC), which may be in response to retrograde menstruation or the presence of endometrial cells undergoing apoptosis [48]. These white blood cells are upregulated and activated to produce pro-inflammatory cytokines and prostaglandins which activate nerves and trigger the upregulation of nociceptive transient receptor potential (TRP) cation channels [92,94]. TRP cation channels have previously been implicated in the progression of chronic endometriotic pain as these channels are expressed in the human endometrium [95]. Recent studies have shown contradictory results that RNA expression levels of various TRP channels do not differ significantly between control groups and endometriosis patients, thus establishing that TRP channel expression does not correlate with endometriosis [95]. Oxidative stress alters the transient receptor potential cation channel, subfamily V, member 1 (TRPV1), which contributes to the generation of pain in inflammatory conditions [96]. Additionally, TRPV1 activation also produces more ROS and increases receptors for tumor necrosis factor alpha (TNF-α), a known inducer of inflammatory hyperalgesia [97,98].

Macrophage secretion products can also recruit other immune cells to the site of “tissue injury” which results in the establishment of a chronic inflammatory milieu in the PC [48,92]. Macrophages have been implicated in other processes such as angiogenesis, promoting cellular proliferation of ectopic lesions, and modulating insulin-like growth factor-1 (IGF-1) which enhances nerve sensitization and increased pain scores in endometriosis animal models [92,94,99]. Forster et al., found that increased levels of macrophage-derived IGF-1 in the PF of women with endometriosis correlated with increased pain scores and neurogenesis [94]. The mechanistic role of macrophages in endometriosis also connects to neuropathic pain and inflammation.

#### 3.1.2. Neurogenic Inflammation

The five cardinal signs of inflammation include dolor (pain), calor (heat), rubor (redness), tumor (swelling). and functio laesa (loss of function). These cardinal signs indicate that when the peripheral nervous system is activated, it directly communicates with and modulates the immune system to have an integral role in inflammation and pain [100,101]. This complex interaction between the immune system and nervous system has led to many pathologies in autoimmune diseases and allergic reactions which suggests that the activated nervous system may play a role in the development of endometriotic pain [101].

Neurogenic inflammation is a specific subtype of inflammation where peripheral neurons such as small C-fiber nociceptors become activated to produce various neuropeptides [102]. In endometriosis, C-fibers are activated by inflammation and other noxious events which stimulate the release of substance P (SP), calcitonin gene-related peptide (CGRP), nitric oxide (NO), and tachykinins into the surrounding microenvironment [103]. These neuropeptides produced by nociceptors can increase localized vascular permeability characteristic of neurogenic inflammation [91,103]. Both SP and CGRP can mediate inflammation. MMP-2 and MMP-9 activities, which are important as MMPs, have been implicated in the remodeling of the ECM which occurs during the progression of endometriosis [104,105]. This has led researchers to conclude that both SP and CGRP contribute to accelerating the progression of endometriosis [104].

Ectopic endometriotic lesions have also been implicated in inducing pain by compressing or infiltrating nerves nearby the lesions [103]. As endometrial lesions do not have an intrinsic nerve supply, these fragments must first migrate and adhere to a novel site to establish a vascular blood supply and become innervated with nerves through the process of “neuroangiogenesis” [93]. This process is remarkable as it shares many characteristics like that of cancer cells including metastasis, invasion, and angiogenesis. Ectopic endometrial lesions may also produce a sensation of hyperalgesia or increased pain sensitivity in the presence of nerve growth factors [103,106]. These studies provide strong evidence that both neurogenic inflammation and pain-inducing ectopic lesions have an intricate role in inducing chronic pelvic pain in endometriosis [103].

Another important mediator for neuropathic pain exhibited in endometriosis patients is via fractalkine (CX3CL1). Fractalkine is a chemokine that has previously been implicated in the neuropathic pain of other inflammatory diseases, but its roles in endometriosis have been less investigated [107,108]. It has been established that CX3CL1 levels in PF and endometrial stromal cells (ESCs) are associated with the progression of endometriosis and invasiveness of ESCs [108]. Liu et al., utilized animal models to evaluate the role of fractalkine in the development of peripheral hyperalgesia in endometriosis. They administered a CX3CL1-neutralizing antibody intrathecally and found that blocking fractalkine reversed hyperalgesia and allodynia [107]. This suggests that this chemokine is essential for the development and progression of neuropathic pain in endometriosis and future studies could focus on targeting CX3CL1 to relieve persistent-neuropathic endometriotic pain [107].

#### 3.1.3. Lipid Peroxides and Pain-Inducing Prostaglandins

It has been well-established that lipid peroxides such as oxidized-low-density lipoproteins (ox-LDLs) are pro-inflammatory and are elevated in the PF of women with endometriosis creating an oxidative stress microenvironment [46,47,48,109]. Oxidized-LDLs increase due to inflammation in the microenvironment, and they function to induce the expression of chemotactic factor monocyte chemotactic protein-1 (MCP-1) [48,78]. MCP-1 is a chemotactic factor produced by macrophages, white blood cells, and endometriotic tissues [110], recruits mononuclear phagocytes to the “site of injury” such as the PC, by binding to chemokine receptors, CCR-2 or CCR-4. Once these monocytes migrate from the vasculature into the PC, they mature into macrophages [110].

As stated previously, macrophages play a vital role in the progression of endometriotic pain. They also do so by releasing pain-inducing molecules such as prostaglandins which function to maintain neuropathic pain in endometriosis [111]. In addition to increased macrophages in the PC, elevated concentrations of the enzymes cyclooxygenase-1 (COX-1) and cyclooxygenase-2 (COX-2) have been apparent in ectopic endometrial lesions in rodent models [112]. Upregulated COX-2 functions to produce the inflammatory mediator, PGE_2_, which plays a vital role in the pathophysiology of endometriosis [91,112]. PGE_2_ contributes to pain hypersensitivity and hyperalgesia by lowering pain thresholds and by enhancing nociceptor sensory fiber excitability by activating EP1-EP4 receptors on nociceptors [91,112]. Our studies showed that the pseudo-prostaglandin-like molecules generated by oxidized LDL are present in the peritoneal fluid of women with endometriosis and are key nociceptive molecules. Our studies further showed that these oxidation-sensitive nociceptive molecules can be blocked by antioxidants [46]. Thus, the dynamic PF of women with endometriosis directly contributes to ongoing pain and inflammation due to the presence of oxidized lipids, increased concentration of macrophages, and pain-inducing molecules [49,92,113]. Due to the complex nature of molecules and pathways at interplay, pain and oxidative stress in endometriosis are rather interconnected and to comprehend the etiology underlying endometriosis, we must investigate mechanisms of both processes. This is particularly necessary so that researchers can develop novel treatment options which target either pain, oxidative stress, or both as a means to treat endometriosis patients.

## 4. Treatments for Oxidative Stress

As oxidative stress is one important etiological mechanism for endometriosis, managing oxidative stress may alleviate some endometriosis-associated symptoms and the clinical severity of this condition. Correcting the imbalance between ROS and antioxidants could be beneficial as this imbalance results in increased oxidative stress which augments disease symptoms, tissue destruction, and the progression of endometriosis [14]. Novel and efficacious treatment options must directly target oxidative stress. For the treatment of other diseases, various antioxidants are frequently prescribed to control oxidative stress [59]. We propose that either a high antioxidant diet or antioxidant supplements may be effective in treating and reducing the severity of endometriosis. Low antioxidant levels in endometriosis may be due to insufficient dietary consumption of antioxidants. A study conducted by Mier-Cabrera et al., found deficient intake of vitamins A, C, and E in addition to zinc and copper when compared to the control group [75]. Women with endometriosis may benefit from participation in a high antioxidant diet [45,113], since this diet could serve to correct the significantly lower levels of antioxidants found in the PF of women with endometriosis [45,113]. Currently, there seems to be a lack of substantial evidence on the efficacy of antioxidant supplementation in counteracting oxidative stress [113,114], which may partially be due to study limitations such as small sample sizes. Some of the antioxidants that have been studied to block oxidative stress in endometriosis are discussed below.

### 4.1. Vitamins C and E

Ascorbic acid or vitamin C (Figure 3) is an essential water-soluble vitamin that supports cellular immunity and functions to reduce oxidative stress [115,116]. Oral vitamin C has been efficacious in reducing NF-κβ activation and the production of inflammatory cytokines TNF-α and IL-6 and can also block cytokine storms when administered intravenously [115]. Due to ascorbic acid’s potential as a powerful antioxidant and anti-inflammatory agent, other studies have suggested its potential benefit in treating endometriosis [46,114,115,117]. Another mechanism for reducing oxidative stress due to iron overload or iron deficiency is by utilizing ascorbic acid’s regulatory function on iron metabolism [118]. Thus, vitamin C and other similar antioxidants may be efficacious treatment options for disorders with underlying iron overload which leads to ferroptosis [118]. Vitamin E, α-tocopherol (Figure 3), is a fat-soluble vitamin that has been utilized in other studies to treat cancer and atherosclerosis, yet the results have been conflicting [117,119]. α-tocopherol has been proposed to treat these conditions as it can inhibit lipid peroxidation, inflammation, and platelet aggregation [119]. As α-tocopherol attenuates the accumulation of lipid peroxides, it has also been implicated in the prevention of ferroptosis [120]. For these reasons, combined vitamin E and C supplementation could be efficacious for the treatment of endometriosis.

Our laboratory previously conducted a clinical trial to evaluate the efficacy of antioxidants vitamin C (1000 mg) and E (1200 IU) daily for 8 weeks, as a combination therapy. It was given to 59 patients who had a history of endometriosis, infertility, and/or pelvic pain [45]. When comparing the two randomly assigned groups of placebo versus antioxidant combination therapy, we found a significant decrease in PF inflammatory markers including IL-6, RANTES, and MCP-1 in addition to a decrease in pain symptoms associated with intercourse (dyspareunia), and menstruation (dysmenorrhea) [45]. MCP-1 is a chemo-attractant that recruits macrophages to the PC, thus decreasing levels of MCP-1 could reduce the secretion of pro-inflammatory cytokines and pain-inducing prostaglandins which are secreted by macrophages [110,111].

Another study conducted by Amini et al., gave women with pelvic pain placebo tablets (control group) or a combination supplement of vitamin C (1000 mg) and E (800 IU) daily for eight weeks [118] and found a significant decrease in the severity of pelvic pain, dyspareunia, and dysmenorrhea in the treatment group after 8 weeks of combined vitamin C and E therapy [118]. These supplemented women had a significant decline in the pro-oxidant marker malondialdehyde (MDA) and ROS levels in comparison to the placebo group [118]. Though some studies have not been able to demonstrate a correlation between vitamin C and E therapy and reduced ROS levels, other studies have demonstrated the efficacy of these supplements as a viable means to reduce oxidative stress markers and pain in endometriosis [45,121]. Future studies need to conduct clinical trials with a larger sample size over an extended time-period.

### 4.2. Vitamin D and Omega-3 Fatty Acids

Vitamin D and omega-3 fatty acids (Figure 3) reduce oxidative stress. Deficiencies in the active form of vitamin D correlate with an increased risk for coronary heart disease and can result in increased mortality rates, whereas omega-3 fatty acids or fish oil supplements have commonly been used to reduce mortality rates in cardiovascular diseases [122,123]. Polyunsaturated fatty acids such as omega-3 fatty acids may regulate antioxidant signaling pathways to lower the incidence of chronic inflammatory diseases, thus they have a potential role for reducing oxidative stress in endometriosis [123]. Currently, the literature is somewhat limited in the use of these agents in relation to endometriosis, yet there is still some evidence of potential therapeutic effects.

Both the inactive form of vitamin D, 25- dihydroxy vitamin D [25(OH)_2_D], and the active form of vitamin D, 1,25-dihydroxyvitamin D [1,25(OH)_2_D], play an essential role in reducing inflammation and intracellular oxidative stress [124]. Vitamin D functions as a strong antioxidant by preventing lipid peroxidation, DNA damage, and protein oxidation all of which can cause cell and tissue damage. Maintaining adequate vitamin D levels will reduce oxidative stress and improve mitochondrial function since a deficiency in vitamin D can affect the mitochondrial electron transport chain to reduce its efficiency, commonly resulting in increased oxidative stress and formation of ROS [124]. Vitamin D and its analogs have been shown to suppress inflammation through inhibiting the activation of NF-κβ in macrophages and by reducing MCP-1 and IL-6 expression [122]. There is also a suggested inverse relationship between the risk for developing endometriosis and plasma levels of 25-hydroxyvitamin D [25(OH_2_)D] [125]. One other mechanism through which vitamin D may reduce oxidative stress is through its iron regulatory function as it acts on hepcidin and ferroportin which are involved in the intestinal absorption of iron [118,126]. Activation and downstream signaling of the vitamin D receptor (VDR) has also been implicated in the inhibition of ferroptosis by regulating the expression of GPX4 and by reducing lipid peroxidation [127].

While some clinical trials have demonstrated the efficacy of vitamin D in improving endometriosis-related pain, other trials have had limited success with vitamin D offering little to no improvement in alleviating pain caused by inflammation and oxidative stress. A double-blind clinical trial by Almassinokiani et al., randomly assigned endometriosis patients experiencing dysmenorrhea and/or pelvic pain 8 weeks after surgical laparoscopy to take either a placebo or a vitamin D (50,000 IU) supplement weekly for 12 weeks [128]. They found no significant differences in visual analogue scale (VAS) scores between the 19 patients in the vitamin D group and the 20 patients in the placebo group for dysmenorrhea (*p* = 0.45) or severity of pelvic pain (*p* = 0.24) [128]. This study was, however, limited by a smaller sample size in both the vitamin D and placebo group.

Researchers have also compared the efficacy of vitamin D and omega-3 fatty acids in reducing oxidative stress, pain, and the sizes of endometriotic lesions. In a randomized, double-blind, placebo-controlled study, female patients suffering from dysmenorrhea experienced a significant reduction in pain using the VAS score after taking vitamin D [129,130]. The participants in this study were randomly assigned to take either a placebo, 1000 mg fish oil, or 2000 IU vitamin D_3_ [129]. There were 20 participants assigned to take fish oil, yet they only experienced a modest improvement in VAS pain [129]. In both the vitamin D and fish oil treatment groups, there was not a significant difference when comparing these interventions to the placebo group [129]. Thus, the literature on omega-3 fatty acids and vitamin D is somewhat inconclusive on their effectiveness in treating endometriosis.

### 4.3. N-Acetylcysteine

N-acetylcysteine (NAC) (Figure 3) is commonly prescribed for the treatment of acetaminophen overdose as it helps to restore glutathione (GSH) levels which are depleted by excessive unconjugated and toxic *N*-acetyl-*p*-benzquinone imine (NAPQI). Recent research implicates NAC as potentially beneficial for the treatment of diseases that involve oxidative stress due to NAC’s powerful antioxidant and free-radical scavenging activity [131]. NAC has many therapeutic targets but its ability to increase intracellular cysteine is of particular importance, since it is the amino acid required for the Xc^−^ antiporter which enables glutathione replenishment during ferroptosis [132].

NAC seems to be a safe and effective treatment option for endometriosis. Porpora et al., conducted an observational cohort study among Italian women with a confirmed diagnosis of ovarian endometriosis and the presence of ovarian endometriomas. After 3 months of treatment, the subjects who were treated with NAC (1.8 g/day) experienced a reduced cyst mean diameter compared to participants in the untreated group who experienced a significant increase in cyst mean diameter [133]. This may be attributed to NAC’s ability to suppress aberrant cell proliferation and excessive activation of inflammatory-related genes [133,134]. Another study by Pittaluga et al., determined that NAC can alter cell behavior by switching cells from a proliferative state to a differentiation state which ultimately decreases cell invasiveness and tissue inflammation, resulting in reduced endometrioma masses [135]. Due to NACs safety profile, it is a viable option for treating progressive and debilitating endometriosis, especially in patients who become pregnant [133].

### 4.4. Melatonin

Melatonin (Figure 3) is a hormone primarily secreted at night by the pineal gland which has been well-established as an antioxidant [136]. Melatonin can act as a free-radical scavenger for hydrogen peroxide and nitric oxide (NO) and it also stimulates antioxidant enzymes [136]. Melatonin has also been shown to decrease lipid peroxidation and markers of ferroptosis [137,138]. Due to these properties, treatment of endometriosis with melatonin should protect lipids, proteins, and DNA from free-radical damage. As previously mentioned, researchers have suggested that MMPs play a vital role in the pathogenesis of endometriosis as these proteolytic enzymes are increased during extracellular matrix (ECM) remodeling of the endometrium [105]. Paul et al., found that after applying melatonin to rats with endometrial lesions, it inhibited protein oxidation and lipid peroxidation as well as reduced proMMP-9 activity [105,136]. This indicates that melatonin can reduce OS in endometriosis by down-regulation of MMPs [136].

Studies also found that melatonin had potent analgesic effects which alleviated intense inflammatory chronic pelvic pain. Pain scores were reduced by 39.8%, dysmenorrhea was reduced by 38.01%, and levels of brain-derived neurotrophic factor (BDNF) were reduced. Their results provided evidence that melatonin reduced the occurrence of analgesic use among endometriosis patients by 80% [103]. These conclusions are important due to the plethora of side effects that can occur with daily or frequent analgesic use such as gastric ulcers resulting from frequent NSAID use. Melatonin could be utilized as an adjunctive option to reduce the amount of pain medication necessary for patients which would ultimately reduce unwanted side effects of pharmacological options.

Other researchers have demonstrated that melatonin receptors, MR1A and MR1B, are expressed in the eutopic endometrium and in ectopic peritoneal lesions. This suggests its potential role in prohibiting estradiol-sensitive endometrial cellular proliferation [139]. Mosher et al., showed that melatonin had the ability to attenuate endometrial cell proliferation in cell culture; however, this does not indicate whether this treatment would be effective in women with endometriosis [139]. Further studies should be conducted in clinical trials to determine how efficacious this therapy would be in downregulating proliferation of endometrial cells in human subjects.

### 4.5. Curcumin

Curcumin is the active ingredient found in the turmeric plant, *Curcuma longa* [140]. Curcumin (Figure 3) has historically been used in Asian medicine as an anti-inflammatory, antioxidant, and anticarcinogenic agent. It functions as an inhibitor of the NF-κβ pathway and acts as an iron-chelating agent [140]. Although curcumin functions as an iron-chelating agent, its effects on ferroptosis are conflicting and still under investigation. Chen et al., demonstrated that a curcumin analog could induce ferroptosis and suppress GPX4 [141]. Another study by Yan et al., utilized curcumin nanoparticles and demonstrated an opposing effect of ferroptosis inhibition [142]. Information on the connection between curcumin and its effects on ferroptosis in endometriosis is still limited and needs further investigation.

Due to curcumin’s anti-inflammatory and antioxidant properties, recent evidence suggests that it may be effective as adjuvant therapy to standard medications in endometriosis [143]. It has an excellent safety profile with little to no side effects [144]. Previously, turmeric has been utilized to treat rheumatoid arthritis, digestive disorders such as inflammatory bowel disease (IBD), pancreatitis, and various types of cancer with its only drawback being poor absorption and bioavailability when taken orally [144,145]. Clinical trials have suggested that 3.6 g of curcumin taken orally for cancer treatment is a therapeutically effective dosage [146]. Various formulations of curcumin have been produced to address the issues of its pharmacokinetics, poor bioavailability, and low solubility when taken orally [142,147]. Some of these formulations include phospholipid complexes, micelles, liposomes, nanoparticles, and micro-formulations which increase the solubility and improve its cellular uptake [147]. As curcumin has aided in the treatment of several inflammatory conditions, it has promising potential as a treatment for endometriosis.

Several clinical trials have supported the use of curcumin supplements for endometriosis due to its antioxidant and pain-reducing therapeutic effects. Signorile et al., utilized a novel dietary supplement including curcumin, omega 3/6, quercetin (Figure 3), parthenium, nicotinamide, and 5-methyltetrahydrofolate to treat endometriosis patients for a 3-month time frame [148]. They observed that this dietary supplement significantly reduced symptoms using the VAS scale in addition to a significant reduction in serum levels of PGE2 [148]. Other studies have demonstrated the ability of curcumin to suppress the growth and proliferation of endometriotic lesions when compared to control groups [149,150]. Zhang et al., proposed that suppression of endometrial cell proliferation was accomplished by reduction of estradiol (E_2_) by curcumin [150]. Although some data exist on the effects of curcumin on oxidative stress regarding other conditions, there needs to be future studies that elucidate curcumin’s role and effectiveness as a therapeutic option for oxidative stress in endometriosis.

Curcumin treatment on human eutopic endometriotic stromal cells (EESCs) inhibited TNF-α induced secretion of IL-6, IL-8, intracellular adhesion molecule-1 (ICAM-1), vascular cell adhesion molecule-1 (VCAM-1), and MCP-1 while also reducing activation of the NF-κβ pathway [143]. TNFR1-dependent signaling induces the transcription of cytokines, pro-inflammatory and anti-apoptotic genes which leads to cellular survival, proliferation, differentiation, and migration in endometriosis. TNF-α binding to its receptor (TNFR1) activates a cascade of events which leads to the activation the NF-κβ pathway. Curcumin may suppress some of the pro-oxidant functions of the NF-κβ pathway by inhibiting the phosphorylation of I kappa B kinase (Iκβ) [27]. For nuclear translocation of the NF-κβ transcription factor to occur, Iκβ must be phosphorylated. Thus, curcumin prevents this nuclear translocation of the NF-κβ p65 subunit and the expression of its pro-inflammatory and anti-apoptotic products [27].

Curcumin’s potential effects of promoting apoptosis are also of importance as endometriosis is characterized by the resistance of endometrial cells to apoptotic-regulated cell death, thus leading to the development and progression of endometriosis as it enables cell survival outside of the uterus [151]. The current literature proposes that inhibition of COX-2 by curcumin in endometriosis cell culture and animal models, may serve to upregulate apoptosis [74]. More research needs to be conducted to fully elucidate the effects and efficacy of utilizing curcumin as a therapeutic option for endometriosis.

### 4.6. Alternative Treatment Options

In addition to the discussed compounds and other currently available pharmaceutical options, other potential treatments for oxidative stress include lifestyle changes and other natural compounds. Several studies have investigated the effects of dietary weight loss and exercise as a viable option for reducing oxidative stress biomarkers. Duggan et al., enrolled 439 overweight/obese postmenopausal women between the ages of 50 and 75 in a 12-month randomized controlled trial to determine the effects of diet and/or exercise on oxidative stress biomarkers [152]. They determined that higher levels of weight loss were significantly associated with greater reductions in oxidative stress byproducts, oxidized-low density lipoprotein (ox-LDL) and F_2_-isoprostanes [152]. Ox-LDL is a circulating marker of oxidative stress, and it results from ROS-mediated damage and lipid peroxidation to cellular membranes [152]. As previously mentioned in this review, ox-LDLs can also induce the recruitment of macrophages to the PC in women with endometriosis, which mediates continuous inflammation and pain [48,110]. Reduction of lipid peroxides from weight loss could be beneficial in improving quality of life for these women with endometriosis. F_2_-isoprostanes, which are also reduced in response to weight loss, are prostaglandin-like pain-inducing compounds derived from the peroxidation of arachidonic acid (AA) [59,87,153]. F_2_-isoprostanes have also been implicated in studies evaluating aerobic training [153]. Aerobic training was found to be successful in reducing oxidative stress but only in those individuals who had higher plasma baseline levels of F2-isoprostanes [153]. These conclusions suggest that regimented weight loss and exercise programs could be useful in reducing specific oxidative stress biomarkers in women with endometriosis.

Dietary interventions have been evaluated as complementary or alternative treatments in other chronic inflammatory disorders. There is insufficient evidence to conclude whether dietary modifications would have a significant impact on chronic inflammatory disorders. Researchers have suggested that patients with rheumatoid arthritis may benefit from adhering to a vegan, Mediterranean, or elimination diet which may reduce inflammatory markers such as erythrocyte sedimentation rate and C-reactive protein and inflammatory cytokines such as IL-1B, TNF-α, and IL-17 [154]. Adhering to an anti-inflammatory or vegan diet may prove to be an effective intervention in endometriosis patients as they have similar immunological processes, elevated pro-inflammatory cytokines, and oxidative stress underlying the condition. There is one study that has suggested the use of a Mediterranean diet in endometriosis patients due to its established antioxidant and anti-inflammatory effects [155]. It has been suggested that women with endometriosis may not consume as many vegetables, dairy products, or omega-3 PUFAs, yet they may consume higher amounts of red meat and trans fats. Thus, it may be beneficial for these women to partake in an anti-inflammatory diet rich in fruits, vegetables, and seafood abundant in flavonoids, carotenoids, and omega-3 fatty acids [156].

Polyphenols are compounds abundant in fruits and vegetables. They are classified as flavonoids such as anthocyanins which have been found to protect cells against oxidative stress due to their antioxidant and anti-inflammatory characteristics [35]. Khanna et al., highlighted the link between consumption of fruits rich in phytochemicals as a way to reduce oxidative stress and inflammation [154]. They determined that anthocyanins found in black rice, black soybeans, and eggplants function to reduce oxidative stress by decreasing MDA and increasing SOD levels [154]. Resveratrol (Figure 3) found in black grapes, berries, peanuts, and red wine can also reduce oxidative stress by scavenging hydroxyl and peroxide free radicals [155,157,158]. Resveratrol’s use as an antioxidant has already been suggested in various other conditions such as cardiovascular disease, metabolic syndrome, and chronic obstructive pulmonary disease (COPD) [157,158,159]. A similar diet or use of supplements could be beneficial for women with endometriosis as they have immunological processes and elevated pro-inflammatory cytokines like that of other chronic inflammatory diseases [160]. Pycnogenol is another plant-derived mixture of polyphenols and procyanidins (bark of the pine tree *Pinus pinaster*) that have both antioxidant and anti-inflammatory properties by inhibiting the NF-kB pathway. When Pycnogenol was compared with a Gn-RH agonist in women with endometriosis, Pycnogenol reduced pain scores over time [161]. Pycnogenol increased the efficacy of oral contraceptives in women with endometriosis [162].

There is now interest in studying microbiome in various diseases including endometriosis [163]. Studies have examined the gut microbiota in relation to the development and treatment of endometriosis [164,165]. Though, Hantschel et al. [166,167] did not find significant changes in gut microbiota in endometriotic mice 21 days after inoculation, other studies in baboons [168] or mice [169] have found that inoculation with endometriosis significantly changed the abundance and diversity of the gut microbiome after 45–90 days, resulting in dysbiosis. The association between gut microbiota and endometriosis was further elucidated in a study by Chadchan et al., who found that oral antibiotics reduced endometriosis progression in mice [170]. Other studies found that pro- or prebiotic supplementation had favorable effects on oxidative stress and inflammation in various disease states, suggesting that alteration of the gut microbiota may be a valid alternative or adjunctive treatment. While studies have shown that the probiotic, lactobacillus, upregulates production of IL-12 and has been shown to decrease endometriosis-related pain and disease progression when given orally [165,171], another study also showed that the gut microbiota-derived species, n-butyrate, inhibited endometriotic lesion growth and inflammation [166].

Future studies need to investigate a potential link between an anti-inflammatory diet and improved oxidative stress in human subjects with endometriosis. Currently, there is insufficient evidence to suggest clinical benefits or reduced symptoms from an endometriosis-specific weight loss or anti-inflammatory diet. Researchers should analyze existing studies on the association between diet and weight loss and reduced oxidative stress biomarkers as a potential therapeutic target or adjuvant therapy for endometriosis.

## 5. Conclusions

**Figure 4:**
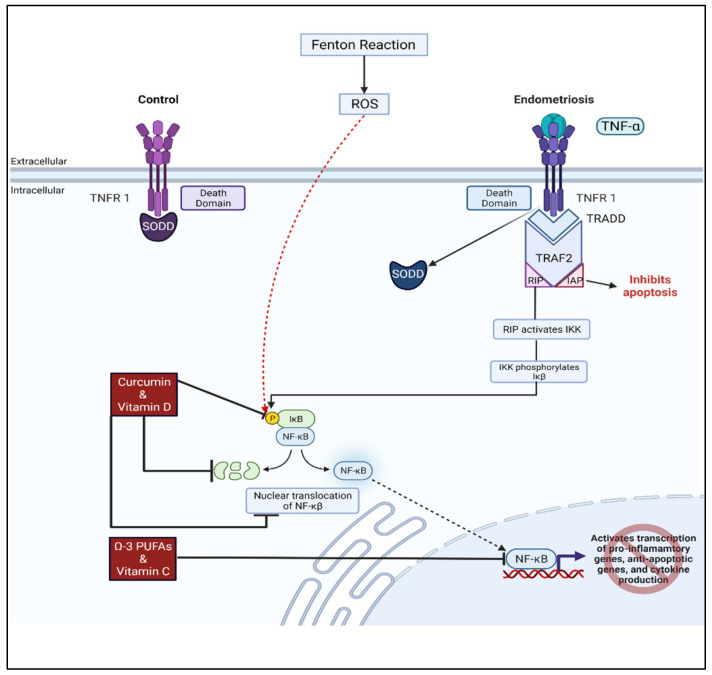
Potential therapeutic options for attenuating oxidative stress and inflammation in endometriosis. Elevated levels of TNF-a present in endometriosis activate the TNFR1-dependent signaling pathway which regulates the transcription of genes that prevent apoptosis and promote inflammation and cytokine production. Upregulation of this pathway leads to cell survival, proliferation, differentiation, and migration in endometriosis in comparison to controls. Oxidative stress resulting in elevated ROS increases the phosphorylation and degradation of IkB leading to nuclear translocation of NF-kB which activates gene expression. Curcumin and vitamin D supplementation may inhibit the phosphorylation of IkB preventing NF-kB nuclear translocation and expression of downstream targets. Omega-3 PUFAs and Vitamin C have the potential for reducing the activity of transcription factor NF-kB. Adapted from “Intracellular Layout—Nucleus and Endoplasmic Reticulum”, by Biorender.com (2022). Retrieved from https://app.biorender.com/biorender-templates (accessed on 18 July 2022).

Current treatment options are not curative for endometriosis as they only address managing various symptoms of the disease. Researchers need to further evaluate the etiology of endometriotic oxidative stress and pain so that future therapeutic options can more effectively target the causes of endometriosis in lieu of simply addressing the symptoms. It is essential to seek out alternative and more natural, biologically active compounds as treatment options for endometriosis as they lack traditional side effects commonly imposed on patients by the currently available pharmaceutical agents. It is imperative to improve the quality of life for women living with this disorder as endometriosis has a high prevalence affecting up to 15% of reproductive-aged women and up to 70% of women experiencing chronic pelvic pain [172]. This review offers evidence that mechanisms underlying oxidative stress and pain (Figure 4) still need further investigation as there are many conflicting results for treating endometriosis with antioxidants and other anti-inflammatory bioactive, non-hormonal and non-addictive agents.

## Data Availability

Not applicable.
